# CENPN Acts as a Novel Biomarker that Correlates With the Malignant Phenotypes of Glioma Cells

**DOI:** 10.3389/fgene.2021.732376

**Published:** 2021-09-27

**Authors:** Hailong Wu, Yan Zhou, Haiyang Wu, Lixia Xu, Yan Yan, Xiaoguang Tong, Hua Yan

**Affiliations:** ^1^ Clinical College of Neurology, Neurosurgery and Neurorehabilitation, Tianjin Medical University, Tianjin, China; ^2^ Department of Neurosurgery, Shijiazhuang Third Hospital, Hebei, China; ^3^ Tianjin Key Laboratory of Cerebral Vascular and Neurodegenerative Diseases, Tianjin Neurosurgical Institute, Tianjin Huanhu Hospital, Tianjin, China; ^4^ Clinical Laboratory, Tianjin Huanhu Hospital, Tianjin, China; ^5^ Department of Neurosurgery, Tianjin Huanhu Hospital, Tianjin, China

**Keywords:** CENPN, biomarker, bioinformatics analysis, glioma, immune infiltration

## Abstract

**Background:** Gliomas are the most common intracranial malignant neoplasms and have high recurrence and mortality rates. Recent literatures have reported that centromere protein N (CENPN) participates in tumor development. However, the clinicopathologic significance and biological functions of CENPN in glioma are still unclear.

**Methods:** Clinicopathologic data and gene expression profiles of glioma cases downloaded from The Cancer Genome Atlas (TCGA) and Chinese Glioma Genome Atlas (CGGA) databases were utilized to determine the associations between the expression of CENPN and clinical features of glioma. Kaplan-Meier and ROC curves were plotted for prognostic analysis. Gene set enrichment analysis (GSEA) and single sample gene set enrichment analysis (ssGSEA) were applied to identify immune-related functions and pathways associated with CENPN’ differential expression. *In vitro* experiments were conducted to investigate the impacts of CENPN on human glioma cells.

**Results:** Elevated CENPN expression was associated with unfavorable clinical variables of glioma patients, which was validated in clinical specimens obtained from our institution by immunohistochemical staining (IHC). The GSEA and ssGSEA results revealed that CENPN expression was strongly correlated with inflammatory activities, immune-related signaling pathways and the infiltration of immune cells. Cell experiments showed that CENPN deficiency impaired cell proliferation, migration and invasion ability and increased glioma apoptosis.

**Conclusion:** CENPN could be a promising therapeutic target for glioma.

## Introduction

Gliomas are the most common intracranial malignant neoplasms, and they present high recurrence and mortality rates (Ostrom et al., 2018). Although multimodal treatments have been developed, glioma patient’s overall survival (OS) is still limited (5 years survival of approximately 5.5%) (OmuroDeAngelis 2013). Molecular markers, such as 1p/19q codeletion status and isocitrate dehydrogenase (IDH) mutations, play valuable roles in tumor formation and progression (Kristensen et al., 2019; Wang et al., 2020). Individually tailored strategies targeting these biomarkers have been adopted in multiple clinical trials, although few have made breakthroughs. Thus, new targets for glioma therapy are urgently needed.

During mitosis, the assembly of kinetochore proteins at the centromere contributes to the accurate segregation of chromosomes (Kops et al., 2005; McKinleyCheeseman 2016). Centromeric Proteins (CENP), containing 18 subtypes, dynamically associate and dissociate onto centromeric chromatin during mitosis with microtubule regulation (CheesemanDesai 2008; WesthorpeStraight 2014; Hoischen et al., 2018). Among the CENPs, CENPN aggregates toward centromeres in the S phase and eventually dissociates in the G2 phase (Foltz et al., 2006; Carroll et al., 2009; Fang et al., 2015). Recognition of the CENPA nucleosome core region by CENPN is essential for proper chromosome division and kinetochore assembly (Carroll et al., 2009). Recently, the relevance of CENPN to the occurrence and progression of different cancers has been proposed. In oral squamous cell carcinoma, CENPN knockdown arrests the cell cycle in the G1 phase, which leads to the suppression of cellular proliferation (Oka et al., 2019). Based on bioinformatics analyses, Rahman et al. reported that CENPN was a prognostic marker for colorectal cancer (Rahman et al., 2019). Moreover, Sarah An et al. revealed that CENPN expression was involved in the malignant progression of breast cancer patients with a history of smoking (Andres et al., 2015). Most recently, CENPN was shown to promote hepatocellular carcinoma cell proliferation and CENPN deficiency was shown to increase radiotherapy-induced DNA damage (Wang et al., 2021). However, the roles of CENPN in glioma have not been clarified.

In the present study, CENPN served as a prognostic biomarker for glioma based on data obtained from public cancer databases and clinical specimens. Moreover, we investigated the potential functions of CENPN in the immune infiltration and immunoregulation of the glioma microenvironment. *In vitro* cell experiments were conducted to verify the reliability of bioinformatics data and provided a reference for future research.

## Materials and Methods

### Dataset Selection

Next-generation sequencing data (HTSeq-FPKM) and clinical variables were obtained from TCGA (Wang et al., 2016). In addition, data for 1,018 glioma cases generated with the Illumina HiSeq platform were obtained from the CGGA (Zhao et al., 2021). Apart from that, a total of 112 glioma cases enrolled from the Department of Neurosurgery (Huanhu Hospital, Tianjin, China) were used for validation.

### Bioinformatics Analysis

In TCGA, the differential expression of CENPN was analyzed using GEPIA, a web database containing abundant normal specimens from GTEx database (Tang et al., 2019). Additionally, GSE16011 (Gravendeel et al., 2009), a dataset from the Gene Expression Omnibus database (GEO), was used for validation. The relationships between CENPN and clinical variables were explored using the beeswarm R package. The survival, survminer and ROC packages were used to plot receiver operating characteristic (ROC) curves and Kaplan–Meier curves.

GSEA was conducted based on the well-established gene sets of c2.cp.kegg.v7.2.symbols.gmt and c5.all.v7.2.symbols.gmt in GSEA 4.0 software. ssGSEA was used to transform the 29 immune signatures into scores for each glioma sample (Subramanian et al., 2005; Bindea et al., 2013; Zhou et al., 2020). Additionally, the CIBERSORT deconvolution algorithm was performed to evaluate the immune infiltration of 22 tumor-infiltrating immune cells (TILs) (Newman et al., 2015). The TIL signatures were downloaded from CIBERSORT (https://cibersortx.stanford.edu/). Relevant R packages were obtained from Bioconductor (http://bioconductor.org/) and CRAN (http://cran.r-project.org/). The bioinformatics analyses were conducted using R software.

### Cell Culture and Transfection

The human glioblastoma cell lines LN229 and U251 were purchased from Beijing Beina Chuanglian Biotechnology Institute and cultured in DMEM containing 10% fetal bovine serum (FBS, Gibco, Invitrogen, CA, United States). The siRNAs targeting CENPN mRNA were produced by GenePharma. The sense sequences of these siRNAs were as follows: siCENPN-i, GCG​UGC​AAG​UAU​CAG​UGA​UTT; siCENPN-ii, GAC​CCU​UUG​UUA​CUC​AAA​UTT; and scramble (siScr), UUC​UCC​GAA​CGU​GUC​ACG​UTT. siRNA transfections were performed using Lipofectamine 2000 according to the manufacturer’s instructions (Thermo Fisher Scientific).

### RNA Extraction and Real-Time PCR

TRIzol reagent was used for RNA extraction, and the SureFireRT kit (06-104; Abgen) was applied for RNA reverse transcription. Then, real-time PCR was conducted on a LightCycler 480 II (Roche). The sequences of designed PCR primers were as follows: GAPDH forward, 5′-CAA​TGA​CCC​CTT​CAT​TGA​CC-3′ and reverse, 5′-GAC​AAG​CTT​CCC​GTT​CTC​AG-3′; CENPN forward, 5′- TGG​ATG​AGA​CTG​TTG​CTG​A -3′ and reverse, 5′- ACTTGCACGCTTTTCCTC -3′. The PCR conditions were 95°C for 5  min, followed by 40 cycles at 95°C for 10  s and 60°C for 1 min. Relative quantification was calculated by the 2^−ΔΔCt^ method.

### Western Blotting and Immunohistochemistry

Cells were lysed using RIPA buffer following the manufacturer’s protocols (Solarbio Co., Beijing, China). Protein (50 μg/lane) was added to the 12% SDS-PAGE gel and then transferred to a membrane (PVDF, Merck Millipore). The protein blots on the membrane blocked by BSA were incubated with antibody against CENPN (1:500 dilution; Affinity Biosciences, Jiangsu, China; DF2315) or β-tubulin (1:500 dilution; CST, United States) overnight. After incubation with HRP-linked goat anti-rabbit IgG (CST, United States) for 60 min, the membrane was exposed to enhanced chemiluminescence reagent.

Immunohistochemistry assays were conducted by the pathology department of Huanhu Hospital. Immunostaining of CENPN was performed using a rabbit polyclonal anti-CENPN antibody (1:500 dilution; Affinity Biosciences, Jiangsu, China; DF2315). The positive slides were recognized by integrated scoring, which was performed independently by two experienced pathologists. The glioma tissues were divided into high and low expression groups based on the expression density of CENPN, as previously reported (Xiao et al., 2015).

### CCK-8, Colony Formation, Wound Healing, Invasion, Apoptosis and Cell Cycle Analysis

5 × 10^3^ cells per well were seeded in 96-well plates and then transfected with siCENPN. Cells were incubated with CCK-8 reagent (K009-500, ZETA) for 30–60 min at room temperature at 0, 24, 48 and 72 h time points. Eventually, the absorbance changes were observed at 450 nm on a molecular devices microplate reader.

For the wound-healing assay, the bottom of plate was scraped by a pipette tip when 100% cell confluence. Then, wound closure (%) was observed at 0 and 24 h by ImageJ. For the Transwell assay, 5 × 10^4^ cells with or without CENPN knockdown were seeded in the upper chamber (8 µm pore size, Corning, Cambridge, United States) coated with Matrigel (BD Biosciences) while 500 µl of medium containing 10% FBS was placed in the lower chamber. Twenty-four hours later, the upper chamber was immersed in 4% paraformaldehyde for 20 min and then subjected to 20 min crystal violet staining. Eventually, the number of penetrating cells was calculated randomly by three fields of view (FOVs). A colony formation assay was conducted to examine the glioma proliferation ability, with 500 cells per well seeded in six-well plates. Fourteen days later, the cells were fixed with 4% formaldehyde and subjected to crystal violet staining.

Cell apoptosis assay (FITC Annexin V Apoptosis Detection Kit, BD Biosciences, United States) was conducted according to the manufacturer’s protocol, as previously described (Zhou et al., 2021). For the cell cycle assay, glioma cells were harvested 48 h following transfection and then fixed with precooled 75% alcohol for 3 h. Subsequently, the cells were incubated with 0.5 ml of PI/RNase reagent (BD Biosciences, United States) at room temperature for 20 min and then analyzed by flow cytometry.

### Statistical Analysis

Cases with incomplete information were removed from the corresponding analysis. Student’s t-test and one‐way analysis of variance (ANOVA) were used to test for significant differences between two groups or multiple groups, respectively. For CENPN protein expression, the chi-square test was employed. Data are presented as the mean ± standard deviation (SD) of at least three independent experiments. *p* < 0.05 was viewed statistically significant. All statistical tests were two-sided. R software v3.6.2. and GraphPad Prism 8 were used for the statistical analyses.

## Results

### Information of Datasets

In the present study, 1833 glioma cases from the TCGA, CGGA and Huanhu datasets were included. The clinical features of these datasets are summarized in Table 1. Pathology reports containing the WHO grade, IDH1 mutation status and other information on the patients in the Huanhu dataset were provided by the Department of Pathology of Huanhu Hospital. The patients in the Huanhu cohort consisted of 44 females and 68 males, and their age ranged from 24 to 82 years old. Detailed information on the patients in the Huanhu cohort is shown in Supplementary Table S1.

**TABLE 1:** Clinical variables of patients in TCGA, CGGA and Huanhu datasets.

Variables	TCGA	CGGA	Huanhu
Total	703	1,018	112
Age			
≥41	263	191	76
<41	407	558	36
Gender			
Male	651	442	68
Female	460	307	44
Grade			
Ⅱ	249	218	33
Ⅲ	265	240	31
Ⅳ	596	291	48
Histology			
Astrocytoma	194	150	24
Oligodendroglioma	191	76	41
Oligoastrocytoma	130	232	
Glioblastoma	596	291	47
Mixed glioma	—	—	—
IDH1 mutation			
Yes	91	410	76
No	34	339	36
1p19q codeleted			
Yes	—	155	43
No	—	594	24
KPS			
<80	151	—	—
≥80	584	—	—
Ki-67 index (%)			
<10	—	—	34
≥10	—	—	78
P53 mutation			
Yes	—	—	105
No	—	—	6
MGMT methylation			
Yes	—	—	86
No	—	—	24

### mRNA Levels of CENPN in the Databases

**FIGURE 1:**
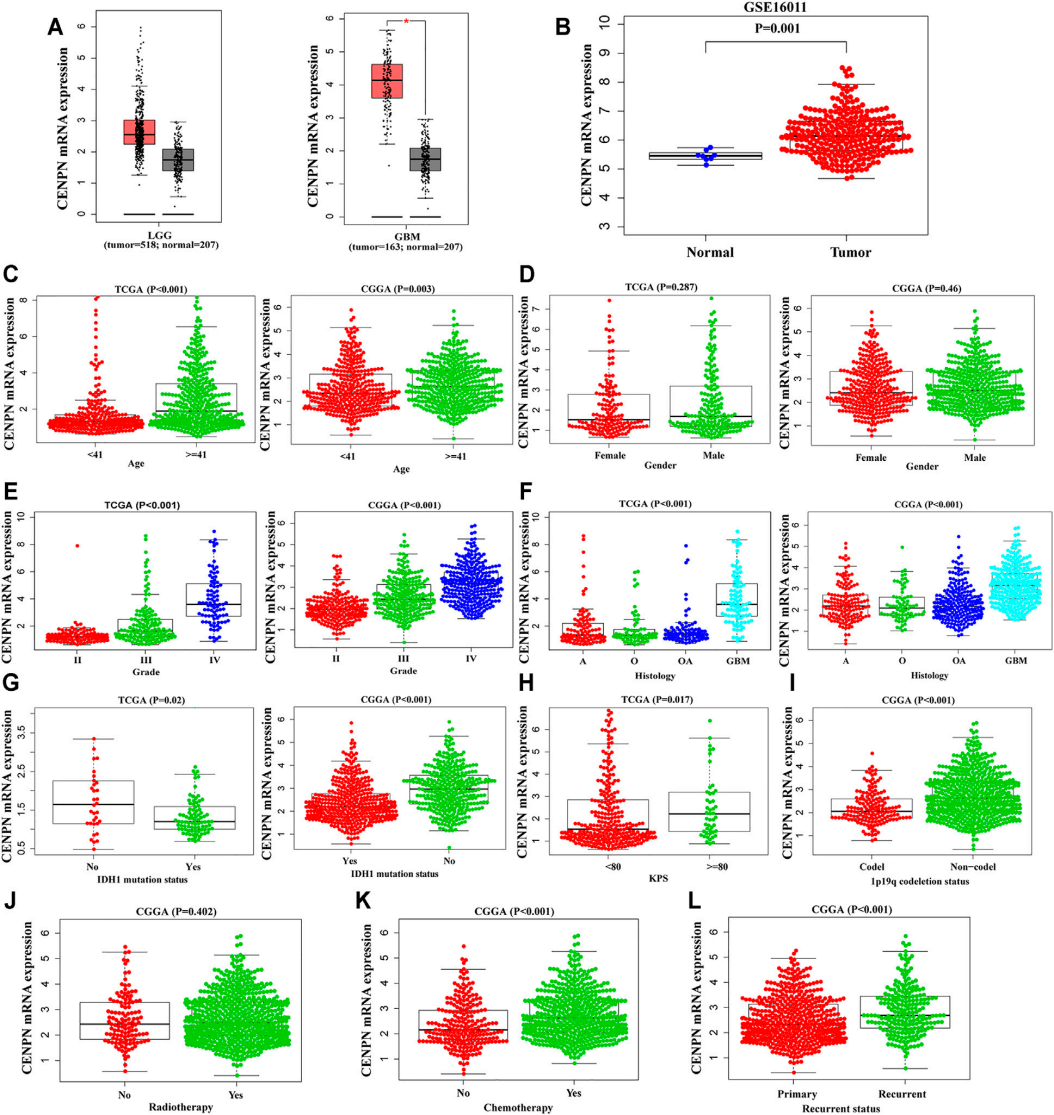
The mRNA expression profiles of CENPN in public databases. **(A,B)** CENPN expression levels in the GEPIA and GSE16011 datasets between tumor and normal tissues. **(C–L)** Associations between CENPN and clinical variables in TCGA and CGGA databases: **(C)** age (year); **(D)** gender; **(E)** WHO grade; **(F)** histological type (A, astrocytoma; O, oligodendroglioma; OA, oligoastrocytoma; GBM, glioblastoma); **(G)** IDH1 mutation status; **(H)** KPS; **(I)** 1p19q codeletion status; **(J)** radiotherapy; **(K)** chemotherapy; and **(L)** recurrence status.

The mRNA expression levels of CENPN in glioma were assessed based on GEPIA and GSE16011, a glioma dataset obtained from the Gene Expression Omnibus database. Compared with the values in normal samples, the CENPN mRNA level was markedly higher in the glioma samples (Figures 1A,B).

### Association Between CENPN and Glioma Clinical Features

To explore the CENPN expression profiles in glioma, the clinical features of glioma patients obtained from TCGA and CGGA were analyzed. As demonstrated in Figures 1C,E–G, CENPN was highly expressed in patients older than 41 years and patients with advanced grades, histopathologies and wild-type IDH1 in both TCGA and CGGA, although no association was found between CENPN expression and gender (Figure 1D). In TCGA, CENPN expression increased with higher Karnofsky Performance Status scores (KPS ≥ 80) (Figure 1H). In CGGA, some other clinical variables (radiotherapy, 1p19q codeletion, chemotherapy and recurrent status) were also analyzed (Figure 1I-L). These results revealed that CENPN upregulation could predict unfavorable glioma.

### CENPN Predicts Worse Survival in Glioma

**FIGURE 2:**
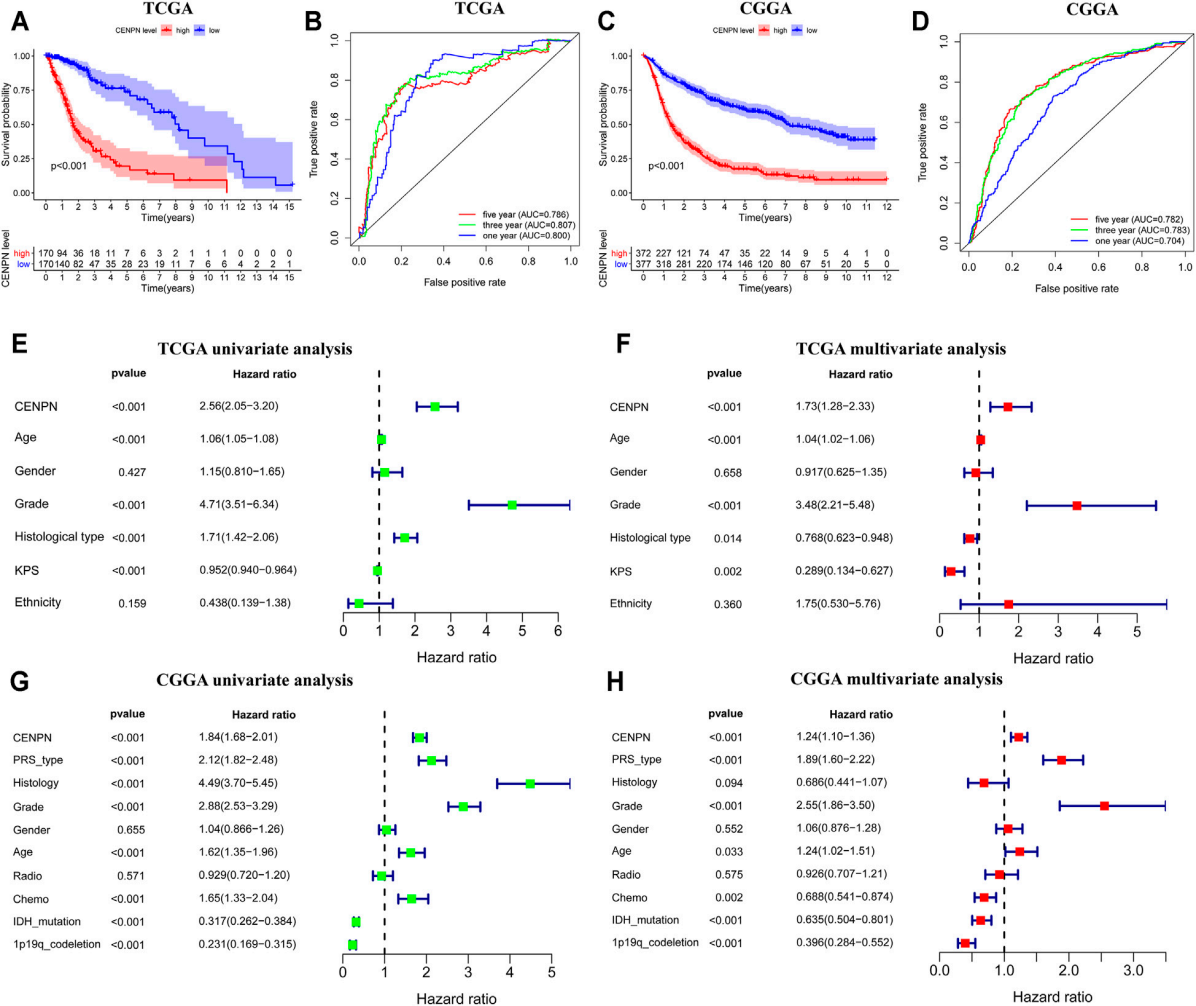
The prognostic values of CENPN in TCGA and CGGA databases. **(A,C)** Kaplan–Meier analysis of OS between groups with different CENPN expression; and **(B,D)** 1, 3- and 5 year ROC curves of OS. Univariate and multivariate Cox regression in TCGA **(E,F)** and CGGA **(G,H)**. Relevant forest plots were plotted.

Kaplan-Meier plots were graphed to estimate the prognostic value of CENPN expression. As depicted in Figure 2A, CENPN high expression was associated with worse survival of patients in TCGA. ROC analyses demonstrated that CENPN could be a fine predictor for patient survival: 1 year (AUC = 0.800), 3 years (AUC = 0.807) and 5 years OS (AUC = 0.786) (Figure 2B). These results were verified in CGGA dataset (Figures 2C,D).

Furthermore, univariate and multivariate Cox regression analyses were employed to assess the independent prognostic roles of CENPN. In TCGA, univariate regression showed that patients with CENPN-high had a shorter overall survival time (hazard ratio (HR): 2.56, 95% CI [2.05–3.20], *p* < 0.001) (Figure 2E). The remaining clinical features associated with OS, such as age, grade and histology, are shown in Figure 2E. The multivariate regression model demonstrated that CENPN was independently correlated with patient OS (Figure 2F, HR = 1.73, 95% CI [1.28–2.33], *p* < 0.001). Moreover, these results were also confirmed in CGGA dataset (Figures 2G,H), indicating that CENPN could be an independent risk factor for glioma.

### CENPN-Related Biological Processes and Immune Activities

**FIGURE 3:**
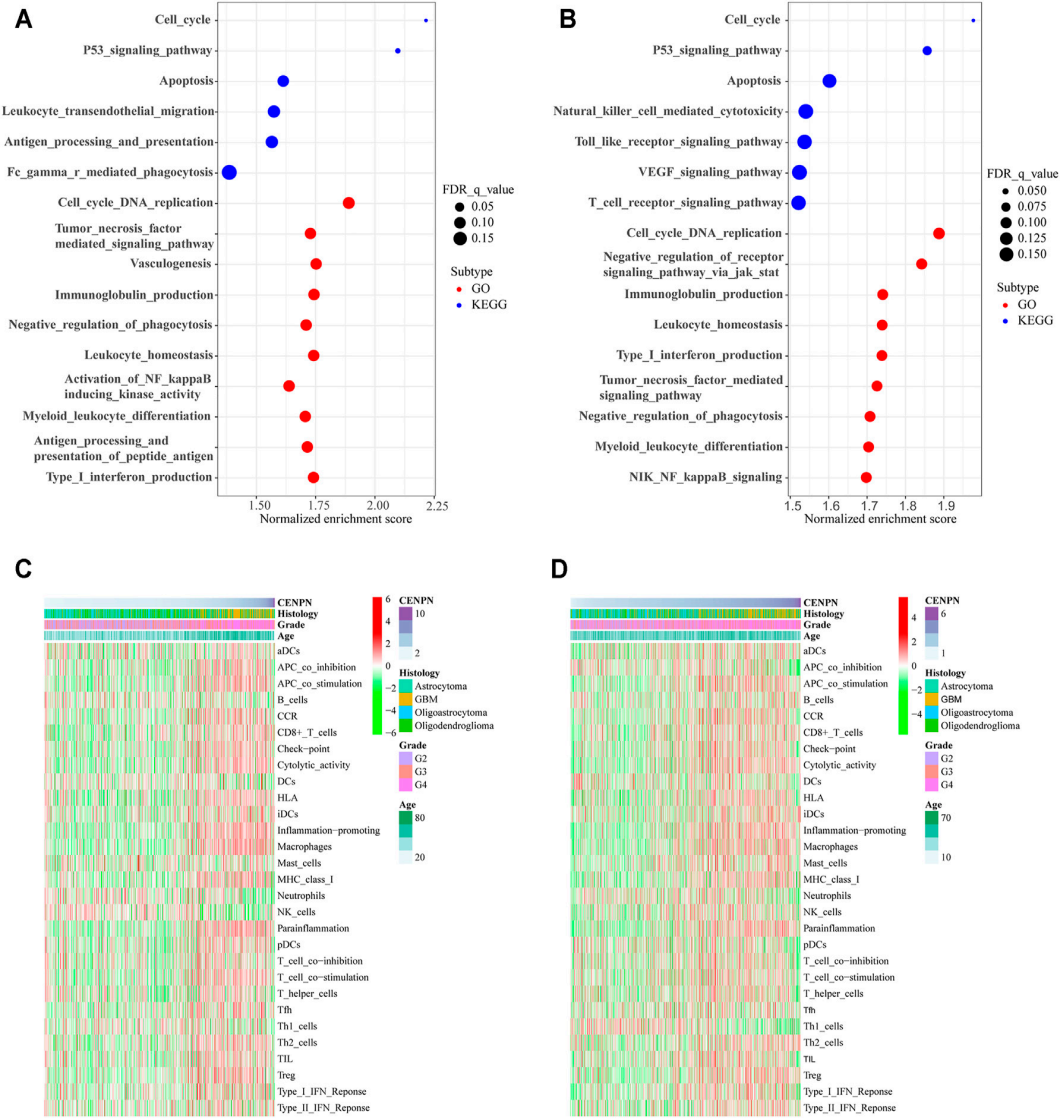
CENPN-related biological processes and immune activities. **(A,B)** GO and KEGG pathway analyses in TCGA and CGGA. A false discovery rate (FDR) < 0.25 was viewed significantly enriched. **(C,D)** Heatmaps based on ssGSEA exhibiting CENPN expression, clinical variables, and 29 immune-related gene sets.

The KEGG analysis based on the GSEA of genes in the high CENPN expression group in TCGA showed that some biological pathways were enriched, including the cell cycle, P53 signaling pathway and apoptosis. Moreover, some pathways related to immunity were also enriched, such as leukocyte transendothelial migration, Fc gamma r-mediated phagocytosis and antigen processing and presentation. The GO analysis indicated that immune-related biological processes, such as immunoglobulin production, leukocyte homeostasis and negative phagocytosis regulation, were enriched in the cohort with CENPN-high (Figure 3A). Similar results were also verified in the CGGA database (Figure 3B). GSEA results suggested that CENPN was associated with multiple molecular mechanisms of glioma development.

To further explore the role of CENPN in the immune microenvironment, ssGSEA was applied to assess the enrichment scores of immune-related pathways, immune cells and immune-related functions grounded on the 29 gene sets derived from previous studies (Subramanian et al., 2005; Bindea et al., 2013; Zhou et al., 2020). In TCGA, the heatmap (Figure 3C) shows that some immune-related functions were positively correlated with CENPN expression. APC coinhibition, APC costimulation, chemokine receptor (CCR), cytolytic activity, immune checkpoint, inflammation promoting, human leukocyte antigen (HLA), parainflammation, major histocompatibility complex (MHC) class I, type I interferon (IFN) responses, T cell coinhibition and T cell costimulation were enriched in CENPN-high patients. Additionally, several immune cell types, such as CD8^+^ T cells, macrophages and regulatory T cells (Tregs), were infiltrated in glioma cases with CENPN-high, while neutrophils, dendritic cells (DCs) and natural killer cells (NK cells) showed opposite trends. Meanwhile, similar results were also obtained in CGGA database (Figure 3D). These results indicated that CENPN has a close relationship with immunomodulation of glioma.

### Evaluation of Immune Infiltration

**FIGURE 4:**
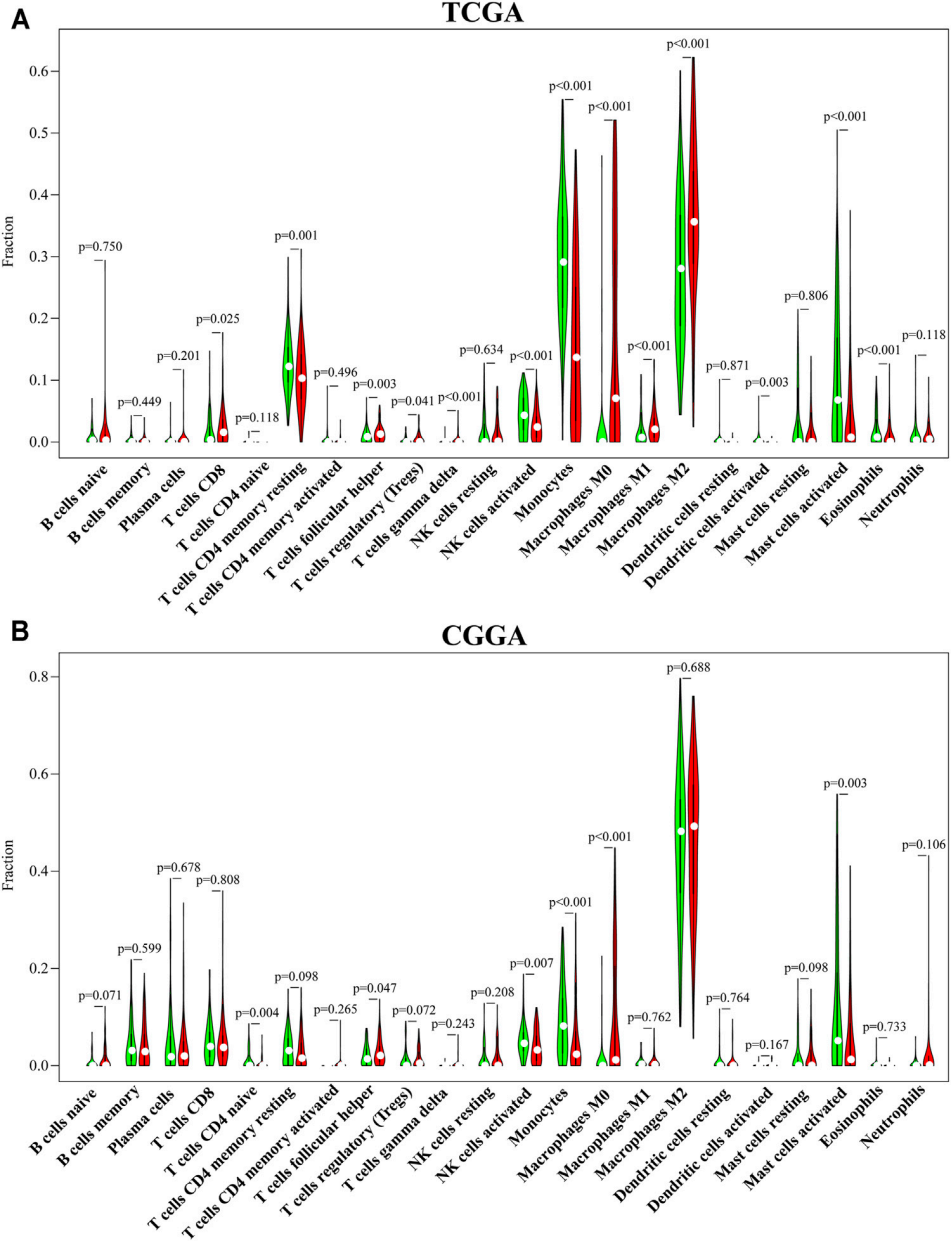
CENPN-related immune infiltration. **(A,B)** The fractions of 22 TILs infiltrating the glioma microenvironment in TCGA and CGGA based on the CIBERSORT algorithm.

To systematically estimate the relationship between CENPN and tumor-infiltrating lymphocytes (TILs), the CIBERSORT algorithm method was employed to infer the fractions of 22 immune cell types in tumor samples based on gene expression profiles. Consistent with the ssGSEA results, CD8^+^ T cells, follicular helper T cells, Tregs, gamma delta T cells, M0, M1 and M2 macrophages in TCGA and follicular helper T cells and M0 macrophages in CGGA were enriched in the CENPN-high group. However, memory resting CD4^+^ T cells, NK cells activated, dendritic cells and mast cells, monocytes and eosinophils in TCGA and CD4 naïve T cells, monocytes, mast cells and NK cells activated in CGGA were enriched in the CENPN-low group (Figure 4). These findings indicated an important role of CENPN in the glioma immune microenvironment.

### Protein Expression Level of CENPN in Glioma Tissues

**FIGURE 5:**
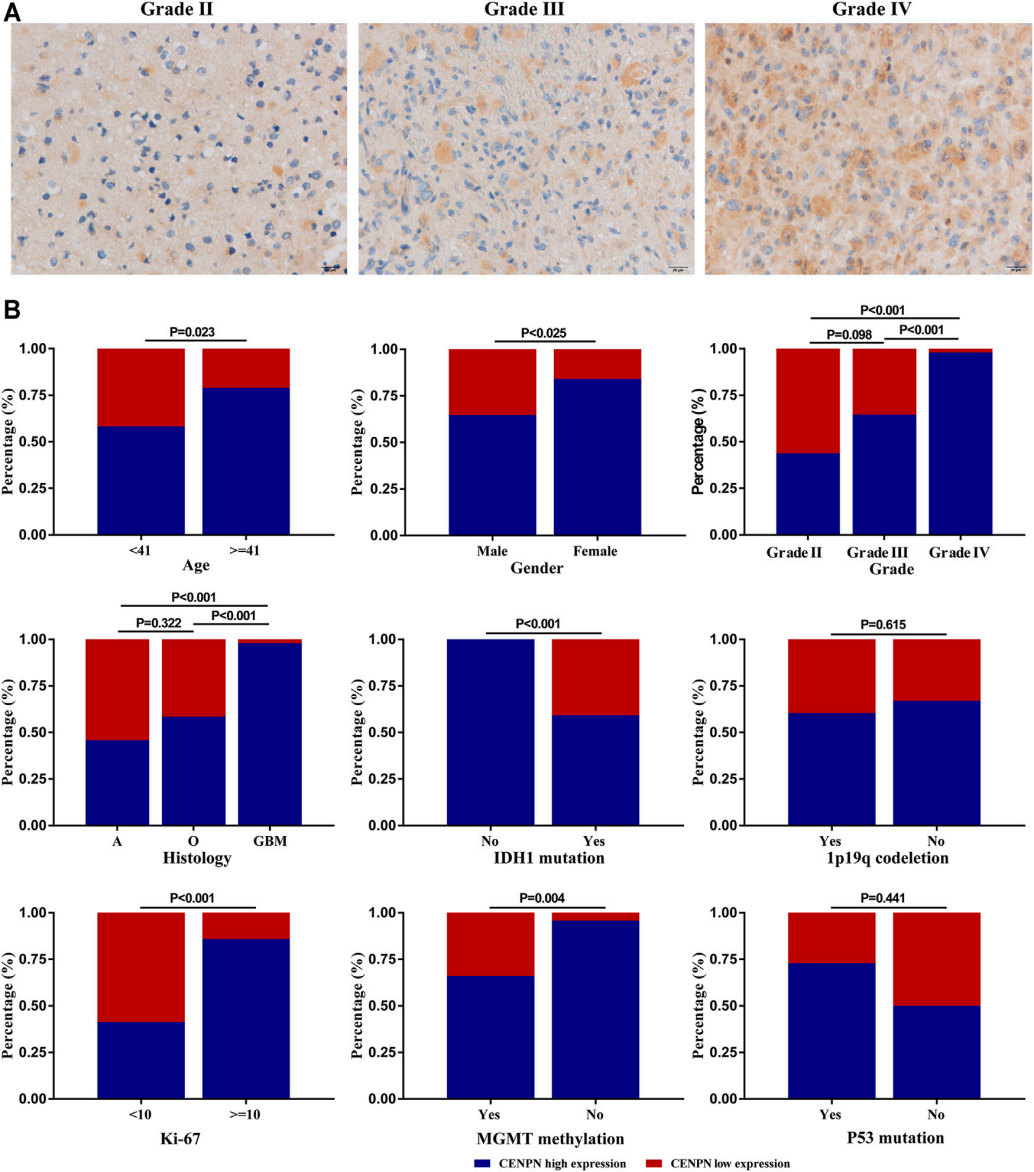
CENPN protein expression in glioma tissues from the Huanhu cohort. **(A)** Representative IHC images of grade Ⅱ, Ⅲ and IV samples. **(B)** Associations between CENPN expression and clinical characteristics in the Huanhu cohort.

Our study further evaluated the protein level of CENPN in 112 glioma specimens by IHC. We found that CENPN was upregulated in 72.3% (81/112) of glioma tissues (Supplementary Table S1). Representative IHC images of samples with grades II, III and IV are displayed in Figure 5A. CENPN expression was positively correlated with some adverse outcome clinicopathological variables, such as age, WHO grade and histology (Figure 5B). However, no correlation was found between CENPN expression and 1p19q codeletion or P53 mutation.

### Biological Functions of CENPN in Human Glioma Cells

**FIGURE 6:**
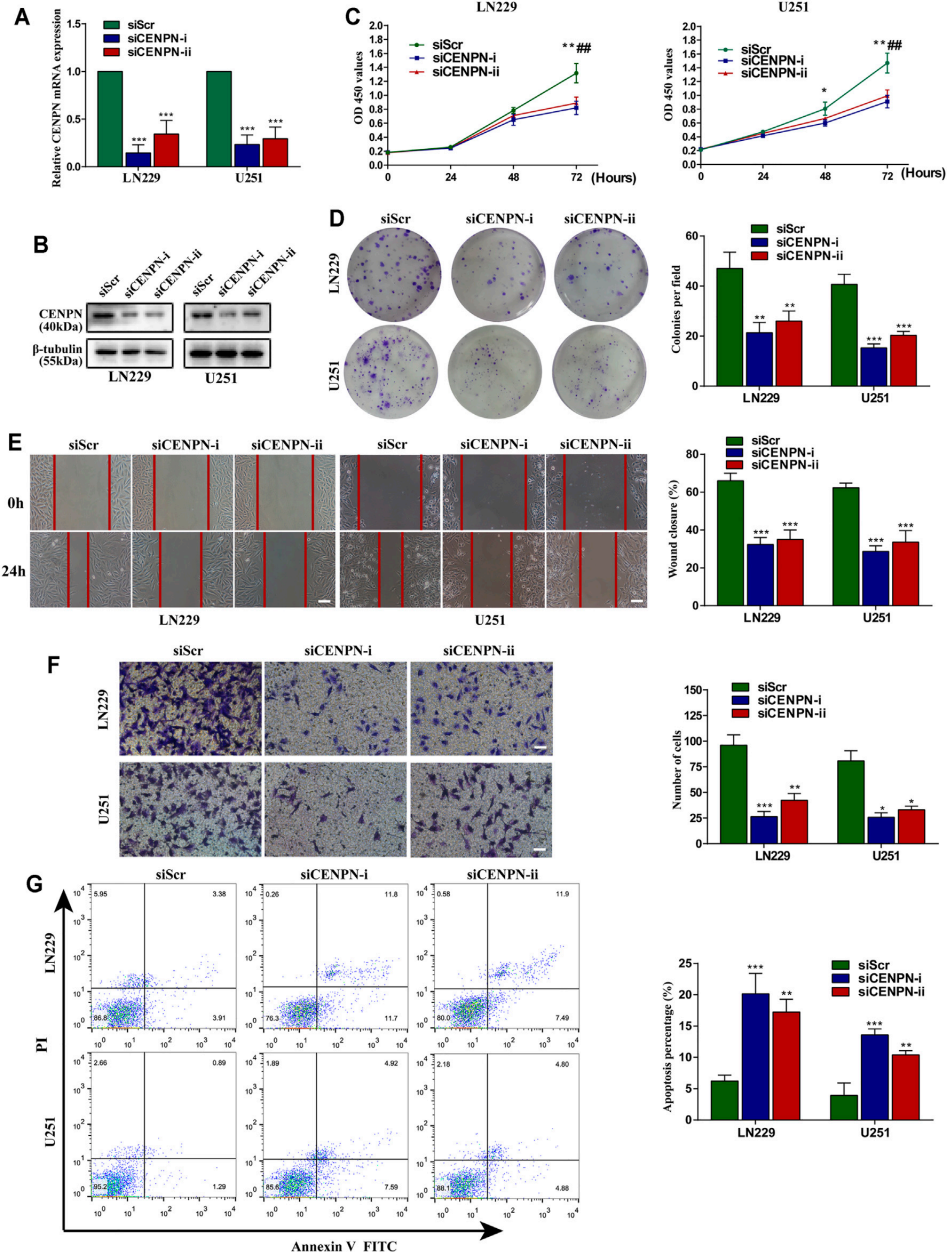
Effects of CENPN knockdown on glioma cells *in vitro*. **(A,B)** Expression of CENPN detected after transfection with siRNA by PCR and western blot. *p < 0.05, **p < 0.01, ***p < 0.001. **(C)** CCK-8 assay was used to estimate the effect of CENPN knockdown on cell proliferation. *p < 0.05, **p < 0.01, ***p < 0.001, siCENPN-i vs siScr group; #*p* < 0.05, ##*p* < 0.01, ###*p* < 0.001, siCENPN-ii vs siScr group. **(D)** Cell proliferation was estimated by colony formation assay. **(E)** Wound-healing assay was used to analyze the migration ability of LN229 and U251 cells. Scale bar, 400 μm. **(F,G)** Invasive ability and the degree of apoptosis were estimated by Transwell assay **(F)** and FITC annexin V **(G)**, respectively. Data are the mean ± SD. *p < 0.05, **p < 0.01, ***p < 0.001, one-way ANOVA. Scale bar, 200 μm.

To explore the functions of CENPN in glioma, LN229 and U251 cells were transfected with two siRNAs (siCENPN-i and siCENPN-ii) to silence CENPN transcription. The CENPN siRNAs resulted in significant declines in both mRNA and protein levels (Figures 6A,B). The CCK-8 assay showed that transfection with siCENPN significantly reduced the proliferation of the cells (Figure 6C). The colony formation assay displayed a similar result (Figure 6D). Furthermore, wound healing and Transwell assays showed that downregulation of CENPN suppressed the migration and invasion ability of glioma cells compared with that in the siScr group (Figures 6E,F). In addition, we found that higher apoptotic percentages were detected in the siCENPN groups than in the siScr groups (Figure 6G). However, the cell cycle assay showed that there were no differences in the fractions of cells in the G0/G1, G2/M, and S phases after CENPN suppression compared with those subjected to siScr (Supplementary Figure S1). These results suggest that CENPN could promote malignant phenotypes of glioma cells *in vitro*.

## Discussion

The process of tumorigenesis is complicated and involves abnormalities in various genes and signaling pathways (Losurdo et al., 2018; Ronchi et al., 2019; Wang et al., 2019). The lack of timely and effective treatments may lead to the malignant transformation of low-grade glioma, aggravating the prognosis of patients (HanahanWeinberg 2000; FernaldKurokawa 2013; Li et al., 2017; Cai et al., 2018). Therefore, it is essential to clarify the specific mechanisms of glioma occurrence and progression for tumor early diagnosis and treatment.

Taking advantage of the substantial data gained from public databases, we performed comprehensive bioinformatic analyses for CENPN expression in glioma. The results showed that upregulated CENPN expression was correlated with advanced clinicopathological parameters (age, grade, histological type and KPS, etc.) and represented an independent prognostic factor of glioma. These results were validated in clinical samples obtained from our hospital. GSEA and ssGSEA revealed that some immune-related pathways and functions were differentially enriched in the group with CENPN-high. The CIBERSORT algorithm was further used to investigate the influence of CENPN on the infiltration of 22 TILs in the tumor microenvironment. In addition, *in vitro* cell experiments were conducted. Knockdown of CENPN impaired the proliferation, migration and invasion and increased the apoptosis of glioma cell lines. These results demonstrated that CENPN could be a promising target in glioma therapies.

The centromere is a chromosome site that contains chromatin and protein complexes that bind microtubules during mitosis that allows chromosomes to be equally separated (WesthorpeStraight 2014). During the mitosis process, many CENPs associate with and dissociate dynamically from centromeres (CheesemanDesai 2008). In humans, CENPA, also called the histone H3 variant, is always present in the functional centromere. Disruption or mutation of CENPA leads to a complete failure in centromere formation (Palmer et al., 1991; CheesemanDesai 2008). Nevertheless, the biological functions of CENPN are not well clarified. CENP-N interacts directly with the centromere-targeting domain of CENPA to facilitate centromere assembly (Tian et al., 2018), and the depletion of CENPN can induce the loss of many other CENPs (Foltz et al., 2006; Carroll et al., 2009). Therefore, CENPN seems to be essential in centromere assembly, similar to CENPA. However, studies have reported that CENPN is not indispensable in sustaining existing centromeres and recruiting kinetochore proteins because CENPN protein levels decrease before mitosis (McClelland et al., 2007; Hellwig et al., 2011). In cancers, Wang et al. (2021) and Oka et al. (2019) reported that CENPN knockdown could arrest cell cycle at the G1 phase in hepatocellular carcinoma and oral squamous cell carcinoma, respectively. However, in this study, significant changes were not observed in the distribution of cell cycle phases in glioma cells subjected to CENPN knockdown. CENPN may not promote glioma cell progression by influencing the cell cycle; therefore, additional cell experiments are needed to clarify its specific functions in glioma.

In the past decade, immunotherapies have gained great success in multiple tumor treatments (Newick et al., 2017; Ronchi et al., 2020). However, the therapeutic effects of immunotherapies in glioma are less than satisfactory (Lim et al., 2018). As previously reported, the immune infiltration profiles of the tumor microenvironment can directly reflect the immune status and predict the outcomes of cancers (Bindea et al., 2013; Cheng et al., 2016). In our study, patients with different CENPN expression levels had differences in immune infiltration. Based on the CIBERSORT algorithm, five immune cells had the same infiltration trends in both the TCGA and CGGA cohorts. Among these immune cells, the proportions of activated NK cells and monocytes were lower in the CENPN-high group than in the CENPN-low group (Figure 4). Activated NK cells are powerful innate immune cells that can help enhance antitumor immunity by releasing perforin and granzyme and secreting various cytokines (Andre et al., 2018). Monocytes have the property of transforming into macrophages and can function as antigen-presenting cells to strengthen adaptive immunity (Jakubzick et al., 2017). Given these findings, CENPN upregulation might promote glioma progression by suppressing the innate immune system. Glioma cells can release multiple immunosuppressive cytokines, such as TGF-β and IL-10, to generate a “cold” tumor microenvironment (Gieryng et al., 2017). Thus, strategies that can reverse the protumor infiltration of immune cells are urgently needed.

Our work presented certain limitations that should be noted. First, the sample size of controls cases in both public databases and clinical cohorts from our institution was small; therefore, more studies with sufficient normal samples must be conducted in the future. Second, CENPN knockdown did not affect the cell cycle distribution, which was inconsistent with previous reports (Oka et al., 2019; Wang et al., 2021). Thus, more glioma cell lines should be adopted for cell biological function assays. At last, functional enrichment analyses revealed that some immune-related functions, immune cells and immune-related pathways were correlated with CENPN expression. The mechanisms by which CENPN may immunomodulate the microenvironment of glioma remain to be understood.

## Conclusion

This study showed that upregulated CENPN expression was associated with adverse clinical outcomes of glioma patients. CENPN is also associated with multiple immune processes and immune cell infiltration. In addition, *in vitro* experiments showed that CENPN deficiency impaired the proliferation, migration and invasion and increased the apoptosis of glioma cell lines. All in all, these findings indicate that CENPN might be a valid therapeutic target for glioma.

## Data Availability

The datasets presented in this study can be found in online repositories. The names of the repository/repositories and accession number(s) can be found in the article/Supplementary Material.
