# Supplementary figures and images for: CENPN Acts as a Novel Biomarker that Correlates With the Malignant Phenotypes of Glioma Cells
Source: Front Genet. 2021 Sep 27;12:732376. doi: 10.3389/fgene.2021.732376 (PMC8502822; doi:10.3389/fgene.2021.732376)

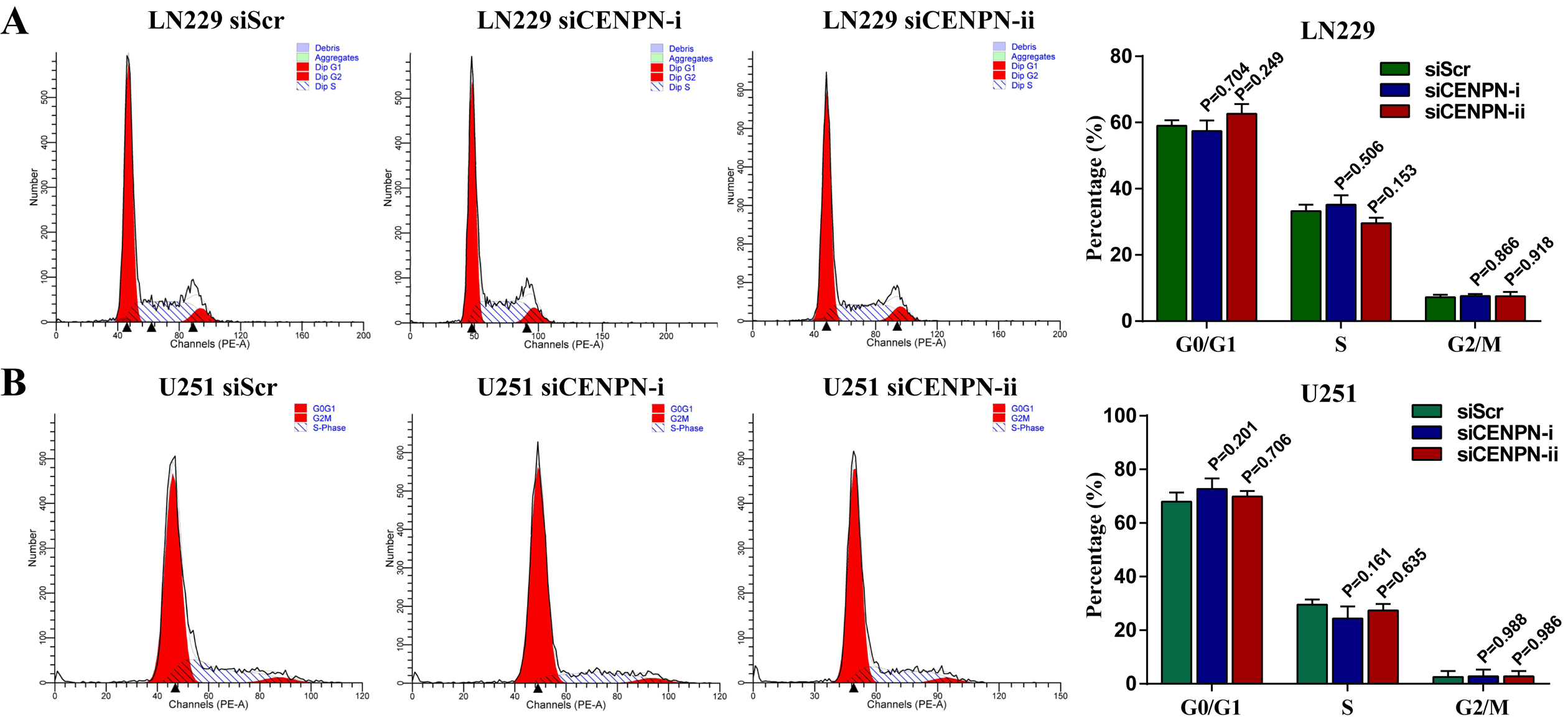

Supplement: Supplementary file 1 [file Image1.TIF]
