# Supplementary material for: CENPN Acts as a Novel Biomarker that Correlates With the Malignant Phenotypes of Glioma Cells
Source: Front Genet. 2021 Sep 27;12:732376. doi: 10.3389/fgene.2021.732376 (PMC8502822; doi:10.3389/fgene.2021.732376)
Supplement: Supplementary file 2 [file DataSheet1.DOCX]

**Table S1. The details of patient characteristics in Huanhu dataset**

| **Number** | **Sex** | **Grade** | **Histology** | **Age (year)** | **Ki-67 index（%）** | **IDH1 mutation** | **1p19q codeletion** | **P53 mutation** | **MGMT methylation** | **CENPN expression** |
| --- | --- | --- | --- | --- | --- | --- | --- | --- | --- | --- |
| 1 | female | Ⅱ | Astrocytoma | 31 | 9.6 | Yes | NA | Yes | Yes | low |
| 3 | female | Ⅱ | Astrocytoma | 34 | 2.6 | Yes | No | Yes | No | high |
| 10 | female | Ⅱ | Astrocytoma | 35 | 3 | Yes | No | No | Yes | high |
| 12 | female | Ⅱ | Astrocytoma | 35 | 7.8 | Yes | No | Yes | Yes | low |
| 13 | female | Ⅱ | Astrocytoma | 38 | 5.6 | Yes | NA | Yes | No | high |
| 17 | female | Ⅲ | Astrocytoma | 55 | 29.2 | Yes | NA | Yes | Yes | high |
| 24 | female | Ⅳ | GBM | 46 | 42.5 | Yes | No | Yes | Yes | high |
| 27 | female | Ⅳ | GBM | 27 | 20 | Yes | No | Yes | No | high |
| 28 | female | Ⅳ | GBM | 50 | 32.6 | Yes | NA | Yes | Yes | high |
| 31 | female | Ⅳ | GBM | 40 | 43.6 | Yes | NA | Yes | Yes | high |
| 33 | female | Ⅳ | GBM | 47 | 25 | Yes | NA | Yes | Yes | high |
| 34 | female | Ⅳ | GBM | 35 | 70 | Yes | NA | Yes | Yes | high |
| 35 | female | Ⅳ | GBM | 41 | 27 | Yes | NA | Yes | Yes | high |
| 38 | female | Ⅱ | Oligodendroglioma | 58 | 9.6 | Yes | Yes | Yes | Yes | high |
| 39 | female | Ⅱ | Oligodendroglioma | 39 | 6 | Yes | Yes | Yes | Yes | high |
| 41 | female | Ⅱ | Oligodendroglioma | 47 | 9.6 | Yes | Yes | Yes | Yes | high |
| 42 | female | Ⅱ | Oligodendroglioma | 41 | 9.2 | Yes | Yes | Yes | Yes | high |
| 43 | female | Ⅱ | Oligodendroglioma | 50 | 4 | Yes | Yes | Yes | Yes | high |
| 45 | female | Ⅱ | Oligodendroglioma | 29 | 7 | Yes | Yes | No | Yes | low |
| 48 | female | Ⅱ | Oligodendroglioma | 38 | 9.2 | Yes | Yes | Yes | Yes | low |
| 50 | female | Ⅱ | Oligodendroglioma | 61 | 5 | Yes | Yes | Yes | Yes | low |
| 56 | female | Ⅲ | Oligodendroglioma | 51 | 40 | Yes | Yes | Yes | Yes | high |
| 57 | female | Ⅲ | Oligodendroglioma | 36 | 13.5 | Yes | Yes | Yes | Yes | high |
| 60 | female | Ⅲ | Oligodendroglioma | 48 | 13.2 | Yes | Yes | Yes | Yes | high |
| 63 | female | Ⅲ | Oligodendroglioma | 55 | 8 | Yes | Yes | Yes | Yes | high |
| 64 | female | Ⅲ | Oligodendroglioma | 37 | 19.8 | Yes | Yes | No | Yes | high |
| 65 | female | Ⅲ | Oligodendroglioma | 39 | 20 | Yes | Yes | Yes | Yes | high |
| 66 | female | Ⅲ | Oligodendroglioma | 51 | 42 | Yes | Yes | Yes | Yes | high |
| 71 | female | Ⅲ | Oligodendroglioma | 58 | 13.2 | Yes | Yes | Yes | Yes | low |
| 72 | female | Ⅲ | Oligodendroglioma | 40 | 18.2 | Yes | Yes | Yes | Yes | low |
| 75 | female | Ⅲ | Oligodendroglioma | 50 | 16 | Yes | Yes | Yes | Yes | high |
| 76 | female | Ⅲ | Oligodendroglioma | 35 | 50 | Yes | Yes | Yes | Yes | high |
| 79 | female | Ⅳ | GBM | 65 | 35.2 | No | NA | Yes | No | high |
| 80 | female | Ⅳ | GBM | 40 | 34.2 | No | NA | Yes | No | high |
| 82 | female | Ⅳ | GBM | 40 | 28.9 | No | NA | Yes | No | high |
| 87 | female | Ⅳ | GBM | 62 | 30 | No | NA | Yes | Yes | high |
| 89 | female | Ⅳ | GBM | 67 | 41.2 | No | NA | Yes | No | high |
| 94 | female | Ⅳ | GBM | 82 | 40 | No | NA | Yes | Yes | high |
| 95 | female | Ⅳ | GBM | 41 | 23.6 | No | NA | Yes | No | high |
| 97 | female | Ⅳ | Astrocytoma | 39 | 16.8 | No | NA | NA | NA | high |
| 100 | female | Ⅳ | GBM | 67 | 40 | No | NA | Yes | No | high |
| 101 | female | Ⅳ | GBM | 67 | 80 | No | NA | Yes | Yes | high |
| 102 | female | Ⅳ | GBM | 59 | 65 | No | NA | Yes | Yes | high |
| 104 | female | Ⅳ | GBM | 59 | 45 | No | Yes | Yes | Yes | high |
| 2 | male | Ⅱ | Astrocytoma | 31 | 4 | Yes | No | Yes | Yes | low |
| 4 | male | Ⅱ | Astrocytoma | 36 | 6 | Yes | No | Yes | Yes | low |
| 5 | male | Ⅱ | Astrocytoma | 39 | 6.2 | Yes | NA | Yes | Yes | high |
| 6 | male | Ⅱ | Astrocytoma | 41 | 4 | Yes | No | Yes | Yes | high |
| 7 | male | Ⅱ | Astrocytoma | 51 | 2.5 | Yes | No | Yes | Yes | low |
| 8 | male | Ⅱ | Astrocytoma | 35 | 6.5 | Yes | No | Yes | Yes | high |
| 9 | male | Ⅱ | Astrocytoma | 54 | 6.8 | Yes | No | Yes | Yes | low |
| 11 | male | Ⅱ | Astrocytoma | 51 | 4.5 | Yes | No | Yes | Yes | high |
| 14 | male | Ⅱ | Astrocytoma | 36 | 3.6 | Yes | NA | Yes | Yes | low |
| 15 | male | Ⅱ | Astrocytoma | 45 | 2.7 | Yes | NA | Yes | No | low |
| 16 | male | Ⅱ | Astrocytoma | 45 | 4 | Yes | NA | Yes | NA | low |
| 18 | male | Ⅲ | Astrocytoma | 24 | 13.2 | Yes | No | Yes | Yes | low |
| 19 | male | Ⅲ | Astrocytoma | 38 | 17 | Yes | NA | Yes | Yes | high |
| 20 | male | Ⅲ | Astrocytoma | 38 | 30 | Yes | No | Yes | Yes | high |
| 21 | male | Ⅲ | Astrocytoma | 36 | 13.8 | Yes | NA | Yes | Yes | low |
| 22 | male | Ⅲ | Astrocytoma | 45 | 9.2 | Yes | No | Yes | Yes | low |
| 23 | male | Ⅲ | Astrocytoma | 56 | 7.5 | Yes | NA | Yes | Yes | low |
| 25 | male | Ⅳ | GBM | 55 | 13.8 | Yes | No | Yes | Yes | high |
| 26 | male | Ⅳ | GBM | 44 | 30 | Yes | No | Yes | Yes | high |
| 29 | male | Ⅳ | GBM | 30 | 45 | Yes | No | Yes | Yes | high |
| 30 | male | Ⅳ | GBM | 37 | 41.2 | Yes | NA | Yes | Yes | high |
| 32 | male | Ⅳ | GBM | 36 | 60 | Yes | No | Yes | Yes | low |
| 36 | male | Ⅱ | Oligodendroglioma | 40 | 8 | Yes | Yes | Yes | Yes | low |
| 37 | male | Ⅱ | Oligodendroglioma | 48 | 9.6 | Yes | Yes | Yes | Yes | low |
| 40 | male | Ⅱ | Oligodendroglioma | 48 | 12.8 | Yes | Yes | Yes | Yes | high |
| 44 | male | Ⅱ | Oligodendroglioma | 54 | 9.2 | Yes | Yes | Yes | Yes | low |
| 46 | male | Ⅱ | Oligodendroglioma | 46 | 8 | Yes | Yes | No | Yes | high |
| 47 | male | Ⅱ | Oligodendroglioma | 36 | 10 | Yes | Yes | Yes | Yes | low |
| 49 | male | Ⅱ | Oligodendroglioma | 49 | 7 | Yes | Yes | No | Yes | low |
| 51 | male | Ⅱ | Oligodendroglioma | 42 | 5 | Yes | Yes | Yes | Yes | low |
| 52 | male | Ⅱ | Oligodendroglioma | 29 | 4.8 | Yes | Yes | Yes | Yes | low |
| 53 | male | Ⅲ | Oligodendroglioma | 50 | 15.8 | Yes | Yes | Yes | Yes | high |
| 54 | male | Ⅲ | Oligodendroglioma | 42 | 10 | Yes | Yes | No | Yes | low |
| 55 | male | Ⅲ | Oligodendroglioma | 33 | 25 | Yes | Yes | Yes | Yes | low |
| 58 | male | Ⅲ | Oligodendroglioma | 61 | 19.8 | Yes | Yes | Yes | Yes | low |
| 59 | male | Ⅲ | Oligodendroglioma | 43 | 16 | Yes | Yes | Yes | Yes | high |
| 61 | male | Ⅲ | Oligodendroglioma | 58 | 18 | Yes | Yes | Yes | Yes | high |
| 62 | male | Ⅲ | Oligodendroglioma | 53 | 15.2 | Yes | Yes | Yes | Yes | high |
| 67 | male | Ⅲ | Oligodendroglioma | 57 | 16.8 | Yes | Yes | Yes | Yes | low |
| 68 | male | Ⅲ | Oligodendroglioma | 46 | 19.6 | Yes | Yes | Yes | Yes | high |
| 69 | male | Ⅲ | Oligodendroglioma | 48 | 22.8 | Yes | Yes | Yes | Yes | high |
| 70 | male | Ⅲ | Oligodendroglioma | 47 | 15.6 | Yes | Yes | Yes | Yes | high |
| 73 | male | Ⅲ | Oligodendroglioma | 44 | 32.8 | Yes | Yes | Yes | Yes | low |
| 74 | male | Ⅲ | Oligodendroglioma | 53 | 15.8 | Yes | Yes | Yes | Yes | high |
| 77 | male | Ⅳ | GBM | 60 | 20 | No | No | Yes | No | high |
| 78 | male | Ⅳ | GBM | 49 | 26.2 | No | NA | Yes | No | high |
| 81 | male | Ⅳ | GBM | 42 | 40 | No | Yes | Yes | Yes | high |
| 83 | male | Ⅳ | GBM | 61 | 40 | No | No | Yes | No | high |
| 84 | male | Ⅳ | GBM | 65 | 32.8 | No | NA | Yes | No | high |
| 85 | male | Ⅳ | GBM | 44 | 28.9 | No | NA | Yes | No | high |
| 86 | male | Ⅳ | GBM | 64 | 35 | No | No | Yes | No | high |
| 88 | male | Ⅳ | GBM | 48 | 39.6 | No | NA | Yes | No | high |
| 90 | male | Ⅳ | GBM | 55 | 25 | No | NA | Yes | Yes | high |
| 91 | male | Ⅳ | GBM | 54 | 35.2 | No | NA | Yes | No | high |
| 92 | male | Ⅳ | GBM | 64 | 28.2 | No | NA | Yes | Yes | high |
| 93 | male | Ⅳ | GBM | 61 | 70 | No | NA | Yes | Yes | high |
| 96 | male | Ⅳ | GBM | 72 | 60 | No | No | Yes | Yes | high |
| 98 | male | Ⅳ | GBM | 50 | 35.6 | No | NA | Yes | Yes | high |
| 99 | male | Ⅳ | GBM | 60 | 70 | No | NA | Yes | No | high |
| 103 | male | Ⅳ | GBM | 39 | 26.8 | No | NA | Yes | No | high |
| 105 | male | Ⅳ | GBM | 64 | 15 | No | No | Yes | No | high |
| 106 | male | Ⅳ | GBM | 52 | 41.2 | No | NA | Yes | Yes | high |
| 107 | male | Ⅳ | GBM | 59 | 35.6 | No | NA | Yes | No | high |
| 108 | male | Ⅳ | GBM | 51 | 36.8 | No | NA | Yes | Yes | high |
| 109 | male | Ⅳ | GBM | 65 | 19.5 | No | NA | Yes | Yes | high |
| 110 | male | Ⅳ | GBM | 66 | 55 | No | NA | Yes | Yes | high |
| 111 | male | Ⅳ | GBM | 68 | 50 | No | NA | Yes | No | high |
| 112 | male | Ⅳ | GBM | 69 | 45 | No | NA | Yes | No | high |

*NA, not available.
